# Supplementary material for: The Systems Biology Research Tool: evolvable open-source software
Source: BMC Syst Biol. 2008 Jun 29;2:55. doi: 10.1186/1752-0509-2-55 (PMC2446383; doi:10.1186/1752-0509-2-55)
Supplement: Additional file 1 — SBRT Archive. An archive of the current version of the Systems Biology Research Tool. [file 1752-0509-2-55-S1.zip › sbrt-1.4.0/doc/developers_guide/api/index-files/index-5.html]

E-Index


|  |  |  |
| --- | --- | --- |
| **Overview** \| Package \| Class \| Use \| **Tree** \| **Deprecated** \| **Index** \| **Help** | |  |
| **PREV LETTER**   **NEXT LETTER** | **FRAMES**    **NO FRAMES**     **All Classes** |  |


A B C D E F G H I J K L M N O P Q R S T U V W X Y Z 

---


## **E**

**EDGE\_FILE** - Static variable in class sbrt.shell.mng.graph\_theory.CycleIdManager: The keyword used to denote the input file containing a set of edges. **EDGE\_FILE** - Static variable in class sbrt.shell.mng.graph\_theory.PathIdManager: The keyword used to denote the input file containing a set of edges. **edges()** - Method in class sbrt.kernel.math.graph\_theory.DirectedGraph: Returns the number of edges in this graph. **ELAPSED\_TIME** - Static variable in class sbrt.shell.mng.AbstractProcessManager: The prefix used to denote the amount of time that has elapsed since an operation was started. **elements()** - Method in class sbrt.kernel.util.BooleanRelation: **EndOfFile** - Class in sbrt.shell.io: This class is used to represent end-of-file (EOF). **EndOfFile()** - Constructor for class sbrt.shell.io.EndOfFile: **entrySet()** - Method in class sbrt.kernel.util.UnmodifiableMap: See Map.entrySet(). **equal(double, double, double)** - Static method in class sbrt.kernel.util.Util: Indicates if the provided values are equal to each other, given the provided tolerance. **equals(Object)** - Method in class sbrt.kernel.fba.AbstractRxn: Indicates if the provided object is equal to this chemical reaction. **equals(Object)** - Method in interface sbrt.kernel.fba.ChemRxn: Indicates if this chemical reaction is equal to the provided object. **equals(Object)** - Method in class sbrt.kernel.fba.ExchangeRxn: Indicates if the provided object is equal to this exchange reaction. **equals(Object)** - Method in class sbrt.kernel.math.AbstractLinearComb: Indicates if the provided object is equal to this linear combination. **equals(Object)** - Method in class sbrt.kernel.math.AbstractLinearComb.Term: Indicates if the provided object is equal to this term. **equals(Object)** - Method in class sbrt.kernel.math.algebra.AbstractMdMatrix: Indicates if the provided object is equal to this matrix. **equals(Object)** - Method in class sbrt.kernel.math.algebra.AbstractSmdMatrix: Indicates if the provided object is equal to this matrix. **equals(Object)** - Method in class sbrt.kernel.math.algebra.IndexPair: **equals(Object)** - Method in interface sbrt.kernel.math.algebra.Matrix: Indicates if the provided object is equal to this matrix. **equals(Object)** - Method in class sbrt.kernel.math.algebra.MatrixElement: **equals(Object)** - Method in class sbrt.kernel.math.algebra.MatrixWrap: Indicates if the provided object is equal to this matrix. **equals(Object)** - Method in class sbrt.kernel.math.geom.ParallelHyperplanes: Indicates if the provided object is equal to this pair of bounding parallelt hyperplanes. **equals(Object)** - Method in class sbrt.kernel.math.graph\_theory.DirectedGraph.Edge: Indicates if the provided object is equal to this directed edge. **equals(Object)** - Method in class sbrt.kernel.math.graph\_theory.Path: Compares the specified object with this path for equality. **equals(Object)** - Method in class sbrt.kernel.math.Interval: Indicates if the provided object is equal to this interval. **equals(Object)** - Method in interface sbrt.kernel.math.LinearComb: Indicates if the provided object is equal to this linear combination. **equals(Object)** - Method in interface sbrt.kernel.math.MathExpr: Indicates if the provided object is equal to this mathematical expression. **equals(Object)** - Method in interface sbrt.kernel.math.Polynomial: Indicates if the provided object is equal to this polynomial. **equals(Object)** - Method in interface sbrt.kernel.math.Polynomial.Term: Indicates if the provided object is equal to this term. **equals(Object)** - Method in class sbrt.kernel.math.stat.comp.MannWhitneyResults: Indicates if the provided object is equal to this results object. **equals(Object)** - Method in class sbrt.kernel.math.stat.comp.TauResult: Indicates if the provided object is equal to this correlation results object. **equals(Object)** - Method in class sbrt.kernel.util.And: **equals(Object)** - Method in class sbrt.kernel.util.BooleanRelation: **equals(Object)** - Method in class sbrt.kernel.util.Or: **equals(Object)** - Method in class sbrt.kernel.util.UnmodifiableCollection: See Collection.equals(Object). **equals(Object)** - Method in class sbrt.kernel.util.UnmodifiableList: See List.equals(Object). **equals(Object)** - Method in class sbrt.kernel.util.UnmodifiableMap: See Map.equals(Object). **equals(Object)** - Method in class sbrt.kernel.util.UnmodifiableSet: See Set.equals(Object). **equals(Object)** - Method in class sbrt.shell.io.EndOfFile: Indicates if the provided object is equal to this end-of-file. **EQUALS** - Static variable in class sbrt.shell.text.SpecialChars: = **EqualsMapEntryFormatV1**<K,V> - Class in sbrt.shell.text: This class is a concrete implementation of `MapEntryFormat`. **EqualsMapEntryFormatV1(SimpleFormat<K>, SimpleFormat<V>)** - Constructor for class sbrt.shell.text.EqualsMapEntryFormatV1: Constructs a new map format using the provided key and value formats. **EqualsMapFormatV1**<K,V> - Class in sbrt.shell.text: This class is a concrete implementation of `MapFormat`. **EqualsMapFormatV1(Formatter<K>, Parser<K>, Formatter<V>, Parser<V>)** - Constructor for class sbrt.shell.text.EqualsMapFormatV1: Constructs a new map format using the provided key and value formats. **EqualsMapFormatV1(SimpleFormat<K>, SimpleFormat<V>)** - Constructor for class sbrt.shell.text.EqualsMapFormatV1: Constructs a new map format using the provided key and value formats. **EQUATION\_FILE\_NAME** - Static variable in class sbrt.shell.mng.fba.NetworkInfoManager: The keyword used to denote the name of the file to which the system of linear equations formed by a fluxome will be written. **EQUATIONS\_FILE** - Static variable in class sbrt.shell.mng.algebra.LinearSolverManager: The keyword used to denote the name of the file containing the system of linear equations. **EQUIV\_RXN\_FILE\_NAME** - Static variable in class sbrt.shell.mng.fba.NetworkInfoManager: The keyword used to denote the name of the file to which stoichiometrically equivalent reactions will be written. **EquivTester**<T> - Interface in sbrt.kernel.math: This interface is used to represent *equivalence testers*. **ERROR\_LOG** - Static variable in class sbrt.ProcessRunner: The name of the file in which errors will be logged. **EuclideanDist** - Class in sbrt.kernel.math.geom.comp: This class is used to compute Euclidean distances. **EuclideanDist()** - Constructor for class sbrt.kernel.math.geom.comp.EuclideanDist: **eval(String, Object)** - Static method in class sbrt.ext.R.R: Evaluates the provided R command. **ExceededLimitException** - Exception in sbrt.kernel.math: This class is used to indicate that a limit has been exceeded. **ExceededLimitException()** - Constructor for exception sbrt.kernel.math.ExceededLimitException: **ExchangeRxn** - Class in sbrt.kernel.fba: This class is used to represent exchange reactions in Flux Balance Analysis. **ExchangeRxn(String)** - Constructor for class sbrt.kernel.fba.ExchangeRxn: Constructs a new exchange reaction for the provided chemical species. **exchangeRxns()** - Method in interface sbrt.kernel.fba.Fluxome: Returns the total number of exchange reactions contained in this fluxome. **exchangeRxns()** - Method in class sbrt.kernel.fba.IrrevRxnFluxomeV1: Returns the total number of exchange reactions contained in this fluxome. **EXCLAMATION** - Static variable in class sbrt.shell.text.SpecialChars: ! **execute()** - Method in class sbrt.shell.mng.AbstractProcessManager: Executes the process this process manager controls. **execute(PrintWriter)** - Method in class sbrt.shell.mng.algebra.LinearCombConverterManager: Executes the process this process manager controls and writes informative messages to the provided print writer. **execute(PrintWriter)** - Method in class sbrt.shell.mng.algebra.LinearSolverManager: Executes the process this process manager controls and writes the results to the provided print writer. **execute(PrintWriter)** - Method in class sbrt.shell.mng.algebra.MatrixConverterManager: Executes the process this process manager controls and writes informative messages to the provided print writer. **execute(PrintWriter)** - Method in class sbrt.shell.mng.algebra.MultipleVectorsConverterManager: Executes the process this process manager controls and writes informative messages to the provided print writer. **execute(PrintWriter)** - Method in class sbrt.shell.mng.algebra.SingleVectorConverterManager: Executes the process this process manager controls and writes informative messages to the provided print writer. **execute(PrintWriter)** - Method in class sbrt.shell.mng.comb.SingleElementUnionManager: Executes the process this process manager controls and writes the results to the provided print writer. **execute(PrintWriter)** - Method in class sbrt.shell.mng.comb.StrictSingleElementUnionManager: Executes the process this process manager controls and writes the results to the provided print writer. **execute(PrintWriter)** - Method in class sbrt.shell.mng.fba.BiggSbmlReaderManager: Executes the process this process manager controls and writes informative messages to the provided print writer. **execute(PrintWriter)** - Method in class sbrt.shell.mng.fba.BiggSbmlTranslaterManager: Executes the process this process manager controls and writes informative messages to the provided print writer. **execute(PrintWriter)** - Method in class sbrt.shell.mng.fba.CatalystDeletionManager: Executes the process this process manager controls, and writes the status or results of this process to the provided print writer. **execute(PrintWriter)** - Method in class sbrt.shell.mng.fba.CdHarFluxVectorManager: Executes the process this process manager controls, and writes the status or results of this process to the provided print writer. **execute(PrintWriter)** - Method in class sbrt.shell.mng.fba.CvCatalystDeletionManager: Executes the process this process manager controls, and writes the status or results of this process to the provided print writer. **execute(PrintWriter)** - Method in class sbrt.shell.mng.fba.CvFbaOptManager: Executes the process this process manager controls, and writes the status or results of this process to the provided print writer. **execute(PrintWriter)** - Method in class sbrt.shell.mng.fba.CvMsFluxIntervalManager: Executes the process this process manager controls, and writes the status or results of this process to the provided print writer. **execute(PrintWriter)** - Method in class sbrt.shell.mng.fba.CvObjectiveFunctionManager: Executes the process this process manager controls, and writes the status or results of this process to the provided print writer. **execute(PrintWriter)** - Method in class sbrt.shell.mng.fba.CvRevRxnFluxIntervalManager: Executes the process this process manager controls, and writes the status or results of this process to the provided print writer. **execute(PrintWriter)** - Method in class sbrt.shell.mng.fba.CvRxnDeletionManager: Executes the process this process manager controls, and writes the status or results of this process to the provided print writer. **execute(PrintWriter)** - Method in class sbrt.shell.mng.fba.CvSimpleFluxIntervalManager: Executes the process this process manager controls, and writes the status or results of this process to the provided print writer. **execute(PrintWriter)** - Method in class sbrt.shell.mng.fba.ExtremeCurrentManager: Executes the process this process manager controls, and writes the status or results of this process to the provided print writer. **execute(PrintWriter)** - Method in class sbrt.shell.mng.fba.FbaOptManager: Executes the process this process manager controls, and writes the status or results of this process to the provided print writer. **execute(PrintWriter)** - Method in class sbrt.shell.mng.fba.FbaSolverManager: Executes the process this process manager controls, and writes the status or results of this process to the provided print writer. **execute(PrintWriter)** - Method in class sbrt.shell.mng.fba.FluxActivityManager: Executes the process this process manager controls, and writes the status or results of this process to the provided print writer. **execute(PrintWriter)** - Method in class sbrt.shell.mng.fba.FluxCapManager: Executes the process this process manager controls, and writes the status or results of this process to the provided print writer. **execute(PrintWriter)** - Method in class sbrt.shell.mng.fba.FluxPlasticityManager: Executes the process this process manager controls, and writes the status or results of this process to the provided print writer. **execute(PrintWriter)** - Method in class sbrt.shell.mng.fba.InitialPointManager: Executes the process this process manager controls, and writes the status or results of this process to the provided print writer. **execute(PrintWriter)** - Method in class sbrt.shell.mng.fba.MetatoolFileWriterManager: Executes the process this process manager controls and writes informative messages to the provided print writer. **execute(PrintWriter)** - Method in class sbrt.shell.mng.fba.MmaNotebookManager: Executes the process this process manager controls, and writes the status or results of this process to the provided print writer. **execute(PrintWriter)** - Method in class sbrt.shell.mng.fba.MsFluxIntervalManager: Executes the process this process manager controls, and writes the status or results of this process to the provided print writer. **execute(PrintWriter)** - Method in class sbrt.shell.mng.fba.MsFluxomeReducerManager: Executes the process this process manager controls, and writes the status or results of this process to the provided print writer. **execute(PrintWriter)** - Method in class sbrt.shell.mng.fba.NetworkInfoManager: Executes the process this process manager controls, and writes the status or results of this process to the provided print writer. **execute(PrintWriter)** - Method in class sbrt.shell.mng.fba.ObjectiveFunctionManager: Executes the process this process manager controls, and writes the status or results of this process to the provided print writer. **execute(PrintWriter)** - Method in class sbrt.shell.mng.fba.PalssonSbmlReaderManager: Executes the process this process manager controls and writes informative messages to the provided print writer. **execute(PrintWriter)** - Method in class sbrt.shell.mng.fba.PalssonSbmlTranslaterManager: Executes the process this process manager controls and writes informative messages to the provided print writer. **execute(PrintWriter)** - Method in class sbrt.shell.mng.fba.RandomConstraintsManager: Executes the process this process manager controls, and writes the status or results of this process to the provided print writer. **execute(PrintWriter)** - Method in class sbrt.shell.mng.fba.RandomEnvManager: Executes the process this process manager controls, and writes the status or results of this process to the provided print writer. **execute(PrintWriter)** - Method in class sbrt.shell.mng.fba.RandomObjectiveManager: Executes the process this process manager controls, and writes the status or results of this process to the provided print writer. **execute(PrintWriter)** - Method in class sbrt.shell.mng.fba.ReverseRxnFluxIntervalManager: Executes the process this process manager controls, and writes the status or results of this process to the provided print writer. **execute(PrintWriter)** - Method in class sbrt.shell.mng.fba.RxnDeletionManager: Executes the process this process manager controls, and writes the status or results of this process to the provided print writer. **execute(PrintWriter)** - Method in class sbrt.shell.mng.fba.RxnRemovalManager: Executes the process this process manager controls, and writes the status or results of this process to the provided print writer. **execute(PrintWriter)** - Method in class sbrt.shell.mng.fba.SbmlWriterManager: Executes the process this process manager controls and writes informative messages to the provided print writer. **execute(PrintWriter)** - Method in class sbrt.shell.mng.fba.SimpleFluxIntervalManager: Executes the process this process manager controls, and writes the status or results of this process to the provided print writer. **execute(PrintWriter)** - Method in class sbrt.shell.mng.fba.SimpleRxnFileTransManager: Executes the process this process manager controls and writes informative messages to the provided print writer. **execute(PrintWriter)** - Method in class sbrt.shell.mng.fba.SlpCycleIdManager: Executes the process this process manager controls, and writes the status or results of this process to the provided print writer. **execute(PrintWriter)** - Method in class sbrt.shell.mng.fba.StoichVariationManager: Executes the process this process manager controls, and writes the status or results of this process to the provided print writer. **execute(PrintWriter)** - Method in class sbrt.shell.mng.fba.SvCdHarFluxVectorManager: Executes the process this process manager controls, and writes the status or results of this process to the provided print writer. **execute(PrintWriter)** - Method in class sbrt.shell.mng.fba.SvInitialPointManager: Executes the process this process manager controls, and writes the status or results of this process to the provided print writer. **execute(PrintWriter)** - Method in class sbrt.shell.mng.fba.WwFluxomeReducerManager: Executes the process this process manager controls, and writes the status or results of this process to the provided print writer. **execute(PrintWriter)** - Method in class sbrt.shell.mng.geom.CdHarManager: Executes the process this process manager controls and writes the results to the provided print writer. **execute(PrintWriter)** - Method in class sbrt.shell.mng.graph\_theory.CycleIdManager: Executes the process this process manager controls, and writes the status or results of this process to the provided print writer. **execute(PrintWriter)** - Method in class sbrt.shell.mng.graph\_theory.PathIdManager: Executes the process this process manager controls, and writes the status or results of this process to the provided print writer. **execute(PrintWriter)** - Method in class sbrt.shell.mng.graph\_theory.UniqueCycleIdManager: Executes the process this process manager controls, and writes the status or results of this process to the provided print writer. **execute()** - Method in interface sbrt.shell.mng.ProcessManager: Executes the process this process manager controls. **execute(PrintWriter)** - Method in interface sbrt.shell.mng.ProcessManager: Executes the process this process manager controls, and writes the status or results of this process to the provided print writer. **execute(PrintWriter)** - Method in class sbrt.shell.mng.stat.CorrelationManager: Executes the process this process manager controls and writes the results to the provided print writer. **execute(PrintWriter)** - Method in class sbrt.shell.mng.stat.KendallTauManager: Executes the process this process manager controls and writes the results to the provided print writer. **execute(PrintWriter)** - Method in class sbrt.shell.mng.stat.MannWhitneyUManager: Executes the process this process manager controls and writes the results to the provided print writer. **execute(PrintWriter)** - Method in class sbrt.shell.mng.utilities.IntervalComparisonManager: Executes the process this process manager controls and writes informative messages to the provided print writer. **execute(PrintWriter)** - Method in class sbrt.shell.mng.utilities.VariableParticipationManager: Executes the process this process manager controls, and writes the status or results of this process to the provided print writer. **execute(PrintWriter)** - Method in class sbrt.shell.mng.utilities.VectorComparisonManager: Executes the process this process manager controls and writes informative messages to the provided print writer. **EXPRESSION\_FILE** - Static variable in class sbrt.shell.mng.utilities.VariableParticipationManager: The keyword used to denote the input file containing mathematical expressions. **ExternalSoftwareException** - Exception in sbrt.ext: This class is used to wrap exceptions thrown by external software packages. **ExternalSoftwareException(Throwable)** - Constructor for exception sbrt.ext.ExternalSoftwareException: Constructs an external software exception. **ExternalSoftwareException(String, Throwable)** - Constructor for exception sbrt.ext.ExternalSoftwareException: Constructs an external software exception. **ExtremeCurrentComputer** - Interface in sbrt.kernel.fba.comp: This interface is used to represent extreme current computers. **ExtremeCurrentManager** - Class in sbrt.shell.mng.fba: This class is used to manage FBA extreme current experiments. **ExtremeCurrentManager()** - Constructor for class sbrt.shell.mng.fba.ExtremeCurrentManager: **ExtremeCurrentManager.Rm** - Class in sbrt.shell.mng.fba: **ExtremeCurrentManager.Rm(ComoFile<String, Double>)** - Constructor for class sbrt.shell.mng.fba.ExtremeCurrentManager.Rm: **ExtremeCurrentProcess** - Class in sbrt.kernel.fba.proc: This class is used to perform extreme current experiments. **ExtremeCurrentProcess(IrrevRxnFluxome, ExtremeCurrentComputer)** - Constructor for class sbrt.kernel.fba.proc.ExtremeCurrentProcess: Constructs a new extreme current experiment.

---


|  |  |  |
| --- | --- | --- |
| **Overview** \| Package \| Class \| Use \| **Tree** \| **Deprecated** \| **Index** \| **Help** | |  |
| **PREV LETTER**   **NEXT LETTER** | **FRAMES**    **NO FRAMES**     **All Classes** |  |


A B C D E F G H I J K L M N O P Q R S T U V W X Y Z 

---
